# Comprehensive Characterization of Micronized Wholemeal Flours: Investigating Technological Properties across Various Grains

**DOI:** 10.3390/foods13010039

**Published:** 2023-12-21

**Authors:** Agata Wojciechowicz-Budzisz, Pavel Skřivan, Marcela Sluková, Ivan Švec, Ewa Pejcz, Michal Stupák, Anna Czubaszek, Joanna Harasym

**Affiliations:** 1 Department of Biotechnology and Food Analysis, Wroclaw University of Economics and Business, Komandorska 118/120 Street, 53-345 Wrocław, Poland; ewa.pejcz@ue.wroc.pl (E.P.); joanna.harasym@ue.wroc.pl (J.H.); 2 Department of Carbohydrates and Cereals, University of Chemistry and Technology Prague, Technická 5, Praha 6-Dejvice, 166 28 Prague, Czech Republic; pavel.skrivan@vscht.cz (P.S.); marcela.slukova@vscht.cz (M.S.); ivan.svec@vscht.cz (I.Š.); 3 Department of Food Analysis and Nutrition, University of Chemistry and Technology Prague, Technická 5, Praha 6-Dejvice, 166 28 Prague, Czech Republic; michal.stupak@vscht.cz; 4 Department of Fermentation and Cereals Technology, Wrocław University of Environmental and Life Sciences, 51-630 Wrocław, Poland; anna.czubaszek@upwr.edu.pl

**Keywords:** wholemeal flour, fine granulation, quality, nutritional composition, rheological properties

## Abstract

With a suitable milling system, it is achievable to produce wholegrain flours that match the granulation and technological properties of refined flours while maintaining a complete nutritional profile. This process also minimizes the generation of additional industrial waste. This study aimed to characterize wholemeal flours with a fine granulation size of less than 160 µm: wheat (MWF), rye (MRF), spelt (MSF), barley (MBF), buckwheat (MBWF), and sorghum (MSGF). For comparison, the plain wheat flour type 530 (T530) was analyzed. The flours were assessed in terms of their chemical compositions and alpha amylase activities (the Falling Number assay), pasting properties (amylograph and a Rapid Visco Analyser (RVA)), water absorption using a farinograph, and technological quality based on their water (WRC) and sodium carbonate solvent retention capacity (SRC) profiles. Among the micronized wholemeal flours, wheat flour (MWF) exhibited the highest nutritional value, greatest water absorption, and highest final gelatinization temperature, but had the lowest energy value, carbohydrate content, water SRC, and sodium carbonate SRC. Wholemeal rye flour (MRF) displayed the lowest nutrient content and the highest amylolytic activity, water absorption, and sodium carbonate SRC. The plain wheat flour type 530 (T530) had the lowest water absorption. Special buckwheat flour (MBWF) showed the highest energy value due to its elevated carbohydrate content, along with the lowest sugar and TDF contents, amylolytic activity, and pasting temperature.

## 1. Introduction

Consumer interest in cereal products with increased nutritional value is constantly growing. The increasing market demand can be met with the introduction of wholemeal bread or pseudo-cereal flours [1]. The consumption of wholegrains proved to have many health benefits and has been associated with a decreased likelihood of developing lifestyle-related disfunctions like type 2 diabetes, metabolic syndrome, and cardiovascular disease [2]. The relation is built upon the abundance and variety of bioactive compounds present in wholegrains, encompassing fiber, vitamins, antioxidants, and phytoestrogens [3].

The primary nutritional benefits of fiber found in the husks of wheat, rye, and various cereals and pseudo-cereals stem from key components, namely cereal beta-glucans and arabinoxylans. Additionally, these brans contain notable accompanying substances, with phenolic compounds being particularly significant, predominantly exhibiting antioxidant properties. Furthermore, some B-group vitamins and minerals are also present in these husks [4].

On the contrary, wholemeal flour can adversely affect the sensory characteristics of products, resulting in a gritty texture and diminished fermentation volume. This, in turn, contributes to an increased firmness of the crumb and alterations in the color, flavor, and taste, ultimately leading to a possibility of a lack of consumer acceptance [5]. Furthermore, the presence of bran particles in wholemeal flour can lead to physical interference, including the dilution of gluten proteins by fiber and competition for water among proteins, fibers, and starches due to the size of the bran particles [6]. When an appropriately fine granulation, comparable to the average granulation of very fine flours (below 150–200 µm) that are commonly used, is attained, the dough’s sorption capacity (water binding) experiences a substantial increase. However, this results in minor changes to its mechanical and sensory properties. Finely ground wholemeal flours with these characteristics can effectively substitute conventional flours, offering the added advantage of significantly higher water binding. Consequently, this leads to a substantial increase in the yield of both the dough and final products [7].

Those are the drawbacks which force producers to search for new solutions in manufacturing wholemeal products with acceptable sensory characteristics [8]. One of the ways to improve the appearance and taste of wholemeal bread is to obtain flour with smaller particles through novel milling processes like impact milling on mills with a vertical axis of rotation. This arrangement occurs due to the fact that the flours are not exposed to such thermal stress as when grinding on conventional impact and roller mills [7]. The main advantage of such special grinding equipment is that the particles of bran show fine granulation, while the degree of mechanical and thermal damage of starch grains in endosperm particles is not higher than in conventional milling. The granulation spectra of these finely ground special wholemeal flours are considerably more homogeneous compared to conventional wholemeal flours, and the mean value of the particle diameter is around 150 μm, depending on the type of raw material.

The addition of such obtained wholemeal flour to the dough results in lower sensory discrepancy in the finished product than in the case of conventionally ground wholemeal flour [7]. Nevertheless, the reduction in particle size also results in a decrease in the size of fibers. Additionally, the starch becomes detached from the protein matrix, as noted by Protonotariou et al. [9]. Noort et al. [10] suggested that the reduction in bran particles creates larger interaction surfaces for proteins and reactive components found in the outer layer of cells. At the same time, it contains more bioavailable dietary fiber (micronization causes a redistribution of fiber components from insoluble to soluble fractions) and other substances as it is a more delicate grinding technique.

The aim of this study was to characterize the chemical composition, rheological properties of pastes and doughs, and the technological quality of special micronized wholemeal flours: wheat, rye, spelt, barley, buckwheat, and sorghum.

## 2. Materials and Methods

### 2.1. Materials

For the experimental part of the study, selected finely ground wholegrain flours from wheat (MWF), rye (MRF), spelt (MSF), barley (MBF), buckwheat (MBWF), and sorghum (MSGF) (with fine granulation < 160 µm) provided by the company Perner Svijany Mill, Ltd. (Svijany, Czech Republic) were used. The flours were obtained through innovative milling using a special mill from Mahltechnik Görgens GmbH (Dormagen, Germany). It is a vertical-axis impact mill, initially not designed for grain processing. However, it has been integrated into a specific production line at Perner Svijany Mill (Svijany, Czech Republic). Remarkably, this mill produces excellent results in the grinding of cereals and pseudo-cereals into wholemeal flours. The grinding process operates on the principle of disintegrating the grist between specifically shaped grinding segments that rotate in multiple levels above each other and a specially modified inner wall shell of the grinding device. Throughout the disintegration process, the material remains suspended in the air stream. This setup allows for the regulation of the grist’s residence time in the grinding chamber, influencing its granulation. This unique arrangement minimizes thermal stress on the flour compared to traditional grinding and roller mills [7].

For comparison, plain wheat flour type 530 (T530) (Perner Svijany Mill, Ltd., Svijany, Czech Republic), obtained by traditional milling, was analyzed.

### 2.2. Methods

#### 2.2.1. Chemical Composition of Raw Materials

The moisture ICC (International Association for Cereal Science and Technology) (No. 110/1) [11]; total protein content—obtained using the Kjeldahl method (ICC No. 105/2) using a Foss Tecator Kjeltec 2400 analyzer (Foss, Hilleroed, Denmark) (MWF, MRF, MSF—N×5.7, MBF, MBWF, MSGF—N×6.25); ash content—with the ICC No. 104/1; fat—with the ICC No. 136; and total dietary fiber (TDF) (Megazyme kit (Ireland)) using Fibertec System (Tecator Foss, Höganäs, Sweden) acc. AOAC (Association of Official Agricultural Chemists) with the 991.43 method [12] were determined for the flours.

The determination of carbohydrate content was calculated from the dry matter and other nutrients/components of the product (dry matter = carbohydrate + fiber + protein + fat + ash). The content of sugars (mono- and disaccharides) was determined chromatographically after the extraction of sugars into aqueous solution. Sugars were measured by anion-exchange chromatography with pulsed amperometric detection (HPAEC-PAD) (electrochemical detector ED50, Dionex, Thermo Fisher Scientific), column CarboPac PA1 (2 mm × 250 mm, Dionex, Thermo Fisher Scientific, Sunnyvale, CA, USA), and pump (model GS50, Dionex, Thermo Fisher Scientific, Sunnyvale, CA, USA) providing a flow rate of 0.25 mL/min at 25 °C (thermostat TCC-100 Dionex, Thermo Fisher Scientific). The mobile phase composition was 16 mmol/L, and an aqueous solution of sodium hydroxide was used for isocratic elution (for 20 min), followed by 20 min of column regeneration in 200 mmol/L to obtain the aqueous solution of sodium hydroxide [13].

The calculation of the energy value of product was based on its nutritional value (from the content of the nutrients, i.e., protein content, digestible carbohydrate content, fiber content, and fat content) and using conversion factors for 1 g of the ingredient. Energy value calculation (kJ/100 g) was carried out as follows: content of protein (g/100 g, *w*/*w*) × 17.2 + content of carbohydrates (g/100 g, *w*/*w*) (without fiber) × 17.2 + content of fat × 37 + content of total dietary fiber × 8.4 [14].

The samples were analyzed at least in duplicate, and the results are expressed on a dry matter (d.m.) basis.

#### 2.2.2. Farinograph Water Absorption

The rheological properties of dough from wholemeal flours were analyzed using a farinograph (Brabender, Duisburg, Germany) (AACC Methods 54-21) [15]. The water binding capacity of wholemeal flours was assessed in a blend with standard T530 wheat flour, which has a known water absorption (with the wholemeal flours comprising 50% by mass) [7]. The measurement was performed in duplicate.

#### 2.2.3. Solvent Retention Capacity Profile

Technological quality of the tested material was determined according to AACC [15] method 56-11, solvent retention capacity (SRC) profile was determined using flour samples of 5 g and the centrifuge Eppendorf 5702 (Eppendorf AG, Hamburg, Germany), and 5.0 g/100 g sodium carbonate in water (sodium carbonate, SC-SRC) and deionized water (water retention capacity, WRC) were also used. The solvent retention capacity (SRC) is expressed as the weight of solvent retained by the flour after centrifugation of the flour suspension with the solvent under the given conditions. It is expressed as a percentage by weight of flour. The result is based on 14% moisture of flour. All the SC-SRC and WRC tests were conducted in duplicate.

#### 2.2.4. Determination of Falling Number

The assessment of alpha-amylase activity was carried out using the Falling Number instrument (type 1400, Perten Instruments, Hägersten, Sweden). The Falling Number of flours was determined according to the Hagberg–Perten method (AACC Method 56-81B) [15]. A suitable laboratory shaker of Polish origin (type SZ Shaker, Biogenet, Józefów, Poland) was employed to generate the suspension. The results of two experiments to determine the Falling Number were validated, with variations not exceeding 5% of their mean value. All tests were conducted in duplicate.

#### 2.2.5. Amylographic Measurements

Properties of pastes made of the flours tested were evaluated using an amylograph (Brabender, Duisburg, Germany) according to AACC methods 22-10. Amylograms obtained were used to read out values of the initial and final gelatinization temperatures (°C) and maximal paste viscosity in amylographic units (AUs). The measurement was performed in duplicate.

#### 2.2.6. Pasting Properties of Flours

The pasting properties of flours were determined using a Rapid Visco Analyzer (RVA) model 4500 (Perten Instruments, Macquarie Park, Australia). Distilled water (25 ± 0.01 g) was added to the flours (3.5 ± 0.01 g) in an aluminum RVA canister. The weights of the H_2_O and flours were adjusted (±0.01 g) to compensate for the differences in moisture content of each sample. In all of the tests, a moisture level of 14% was maintained, resulting in a relatively high solid percentage. Clumping was prevented by stirring with a plastic paddle after which pre-programmed profiles were initiated. The profile for flour was used to capture rheological information (RVA curves); time was 16 min. Each suspension was kept at 50 °C for 1 min and then heated to 95 °C at 12.2 °C/min (over 4.5 min) and held for 2.0 min at 95 °C. It was then cooled to 50 °C at 11.8 °C/min (over 3.5 min) and kept for 5.0 min at 50 °C. All the RVA tests were conducted in duplicate.

### 2.3. Statistical Analysis

The results presented are mean values ± standard deviation (SD). Statistical analysis such as one-way ANOVA was carried out using Statistica 13.3 (StatSoft, Kraków, Poland). Significant differences (*p* ≤ 0.05) between the mean values were determined using Duncan’s Multiple Range Test.

## 3. Results and Discussion

### 3.1. Chemical Composition of Raw Materials

The chemical composition and energy value of the micronized wholemeal flours and control flour T530 are presented in Table 1. Among the analyzed flours, the highest moisture was found in T530, MBWF, and MSGF, and the lowest was found in MWF and MSF. Very similar results for wholemeal finely granulated flour were reported by Skřivan et al. [7]. In the study by Protonototariou et al. [9], micronized wheat flour had lower moisture than store-bought wheat flour, as found in our studies. Different grain treatments during micronization affected the moisture content of the flours, causing them to dry out as a result of both the friction and pressure exerted during the milling process [9]. These observations were confirmed by Xu et al. [16] in their research on the application of airflow ultrafine grinding technology on tartary buckwheat. The highest energy values were found in MBWF and MSGF, which resulted in the highest carbohydrate contents and high fat contents in these flours. The lowest amounts of energy were provided by MWF, MRF, and T530. In our study, we obtained lower results for MWF’s carbohydrate values than Kang et al. [17]. The highest total protein contents were found in MWF and MSF, and the lowest was found in T530. The protein content increases with the increase in flour extract [18], which is why whole-wheat flours have higher protein contents than light flour. A reduced protein content in straight-grade flour could result from the elimination of the bran and aleurone layer, both of which contain a significant amount of protein [18]. Our results are higher than those obtained by Skřivan et al. [7] and Kang et al. [17], but lower than those obtained for micronized wholemeal wheat flours by Protonototariou et al. [9]. The wholegrain spelt wheat flours obtained in the laboratory by Sinkovič et al. [19] had higher protein contents than the MSF we tested. The total ash content serves as an indicator for evaluating the overall inorganic content in food samples, providing insight into the quantity of minerals present [20]. The highest content of ash and TDF was found in MWF. The lowest content of ash was found in T530, which corresponds to this type of wheat flour, and the lowest amount of TDF was obtained for MBWF. The amount of fiber in buckwheat flour varies depends on the grain processing method, and without resistant starch, it is most often in the range of 3 to 6% [21]. Furthermore, Niu et al. [22] discovered that examining the impact of superfine grinding on the quality attributes of whole-wheat flour revealed significant alterations. The grinding treatments were observed to bring about physical transformations in starch granules, such as a decrease in the starch crystal area and an elevation in starch damage. These alterations contributed to modifications in the inherent properties of the starch, including, perhaps, starch resistance. Skřivan et al. [7] obtained lower ash and TDF contents for wheat, higher contents for rye, and similar contents for spelt wholemeal finely granulated flour. In the case of buckwheat flour, the TDF content was more than double that obtained in our research. Protonototariou et al. [9] and Kang et al. [17] obtained lower ash and TDF contents than those in our observations. The highest content of sugars was recorded for MRF, and the lowest was recorded for T530. The commercial wholemeal rye flour analyzed by Makran et al. [23] was characterized by higher moisture, carbohydrate, and dietary fiber contents, a much lower protein content, a lower fat content, and an identical ash content than the MRF analyzed in our studies. In our study, MBF was characterized by lower moisture, fat, ash, and carbohydrate contents, and a higher protein content than wholegrain barley flour obtained in the laboratory by Hussein et al. [24]. MSGF contained the highest amount of fat among the assessed flours, but this value was lower than that obtained by Rumler et al. [25] for wholemeal sorghum flours obtained on roller and stone mills. However, the TDF content was similar.

### 3.2. Farinograph Water Absorption

The calculated farinographic water absorption of the analyzed flours is shown in Table 2.

A farinographic analysis is commonly employed in the bakery industry to ascertain the necessary water absorption needed to achieve the optimal consistency of dough. The MWF was characterized by the highest water absorption. The lowest water absorption was found in T530. The water absorption of wholemeal flours is understandably higher, primarily attributed to their composition, which includes a higher proportion of biopolymers, particularly polysaccharides with hydrocolloid properties [7]. Lower water absorption values for jet-milled wholemeal wheat flour were obtained in the studies by Protonotariou et al. [26]. But they also observed that the water absorption of all flours fluctuated from 73.70 to 83.55% and increased significantly as the particle size of the flours decreased. As the particles fracture into smaller pieces, their specific surface area per unit weight expands, consequently enhancing water absorption [26], while an elevated fiber content leads to increased water absorption, which would be confirmed in the case of MWF with the highest TDF content. Similarly, in the study by Both et al. [3], the water absorption of micronized whole-wheat flour increased by approximately 5.0% with a reduced particle size, but its values were lower than those obtained in our studies.

### 3.3. Solvent Retention Capacity Profile

While earlier findings pertain to the physical effects of the disintegration process, the SRC method is designed to provide a preliminary depiction of the microstructure state of the endosperm post-disintegration. The water retention capacity (WRC) and sodium carbonate retention capacity (SC-SRC) values for the examined flours are detailed in Table 3.

Within the limitations of this method, it can be asserted that the WRC aligns with increased farinographic water absorption. In our study, the most noteworthy values were those of SC-SRC, correlating with the degree of starch damage. Among the analyzed flours, MRF was characterized by the highest WRC and SC-SRC, which may indicate a higher degree of starch damage. The lowest WRC was found in T530, which corresponds to the lowest water absorption result. Švec et al. [27] obtained much higher WRC and SC-SRC values for commercial wheat flour than us. However, MWF was rated by the lowest SC-SRC, but this value is within the range of 80–90%, which is recommended by the U.S. Wheat Associates [28] for bread flour. In the case of the mentioned flours, Skřivan et al. [7] obtained higher values of WRC and SC-SRC.

### 3.4. Determination of the Characteristics of the Starch–Amylase Complex Using the Falling Number and Amylograph

The values of the Falling Number and amylolytic determination of the analyzed wholemeal flours are listed in Table 4.

The assessment of the Falling Number represents a technique for gauging α-amylase activity, yet it does not quantify the enzyme’s quantity [15]. A decreased Falling Number indicates a shorter time of the piston to descend into the gruel, signifying elevated α-amylase activity. Nonetheless, an excessive level of α-amylase activity can lead to complications in bread baking, manifesting as sticky dough, discoloration, adhesive crumbs, or challenges in mechanical processing. MWF was characterized by the highest final gelatinization temperature. Among the analyzed flours, MRF was characterized by the lowest Falling Number, and the lowest initial and final gelatinization temperatures, which may indicate higher α-amylase activity, a higher degree of starch damage, and a large surface area available for the site of enzyme activity. MBWF had the highest Falling Number and maximum viscosity (AU). There is a concept indicating that the resistance to degradation in buckwheat starch granules is likely attributed to their structural differences compared to wheat starch granules, and buckwheat may contain one or more compounds, potentially protein or more enduring substances like tannins and phytic acid, that inhibit α-amylase [21,29]. The results of the present study showed a lower Falling Number for MWF than in the study by Kang et al. [17], suggesting that MWF had higher α-amylase activity than wheat flours produced using a hammer and jet mill. However, in the study by Hussein et al. [24] a lower Falling Number was recorded for wholemeal barley flour obtained in the laboratory than for MBF in our studies. MSF was characterized by the lowest maximum viscosity. In the study by Skřivan et al. [7], lower water absorption levels of wheat, rye, and spelt flour, and the same for buckwheat flour, were noted. However, in the case of the mentioned flours, Skřivan et al. [7] obtained higher Falling Number values (except for buckwheat flour, where the sample weight was reduced). The same authors [7] obtained similar values in the amylolytic determination of the analyzed finely ground wheat and rye wholemeal flours and obtainedslightly higher values for spelt and significantly higher gelatinization temperatures for buckwheat. However, we obtained a much higher maximum viscosity.

### 3.5. Pasting Properties

**Figure 1:**
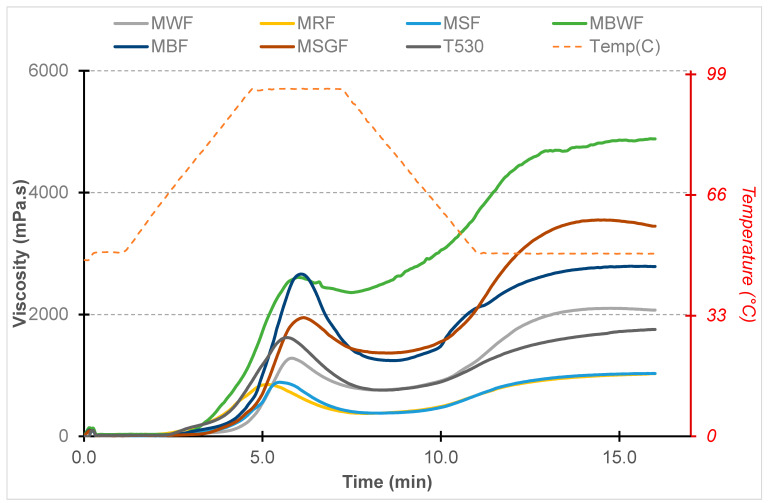
RVA viscograms of analyzed flour samples. MWF—wholemeal wheat flour; MRF—wholemeal rye flour; MSF—wholemeal spelt flour; MBF—wholemeal barley flour; MBWF—wholemeal buckwheat flour; MSGF—wholemeal sorghum flour; T530—plain wheat flour type 530.

A pasting profile analysis is commonly employed to document alterations in the viscosity of starch aqueous suspensions as they undergo controlled heating and cooling processes. The pasting characteristics of flours serve as indicators for specific aspects of flour quality, including starch swelling, retrogradation, and gelatinization [30]. The results are presented in Table 5 and Figure 1.

The highest pasting temperature was characterized by MWF, and the lowest temperatures were observed for MBWF and T530. Kang et al. [17] obtained much lower values of pasting properties of finely ground wheat flours for MWF than us. The highest value of peak time was characteristic for MBF and MSGF, and the lowest value was observed for MRF. Peak viscosity denotes the highest viscosity reached when starch granules are fully expanded to their maximum extent. The RVA diagram of MBF and MBWF displayed the greatest peak viscosity. However, MRF and MSF had the lowest peak viscosity values. The final viscosity is an index reflecting the ability of starch pastes to form networks, and its value largely depends on the rearrangement of soluble amylose during cooling [31]. When talking about holding viscosity, final viscosity, and setback, it should be mentioned that the highest values of these properties were found in MBWF, and the lowest were found in MRF and MSF. The highest breakdown viscosity was found for MBF, indicating the worst paste stability among all flours, and the lowest was found for MBWF. The results of this study regarding the pasting properties of MBWF were consistent with those of Cheng et al. [32]

## 4. Conclusions

In this study, micronized wholemeal bread and non-bread flours were assessed in terms of their chemical compositions, rheological properties, amylolytic activities, pasting properties, and technological quality. Wholegrain flours have high nutritional value, while micronized flours are additionally characterized by unique technological properties. These flours can be an addition to or the basis for baked goods with softer and less gritty textures. The use of these flours can lead to improved baking properties, such as increased volume, a better crumb structure, and improved moisture retention in the final product, which can lead to longer-lasting freshness. Additionally, the finely ground process can enhance the bioavailability of nutrients in the flour.

It was found that MWF was characterized by the highest nutritional value, including the TDF content, which resulted in the highest water absorption and the lowest energy value. The MRF had the lowest nutrient content and the highest amylolytic activity. The highest energy value, which results from the highest carbohydrate content, the lowest TDF content, amylolytic activity, pasting temperature, and breakdown, were found in the MBWF.

After testing micronized flours, the next step is to use these flours for the production of sourdough or, in the longer term, in the production of various nutritional matrices, such as bread, cookies, or pasta.

## Figures and Tables

**Table 1:** Chemical composition and energy value of flours.

Flour Type	Moisture (%)	Energy Value (kcal/100 g)	Total Protein Content (g/100 g d.m.)	Ash (g/100 g d.m.)	Fat (g/100 g d.m.)	Carbohydrates (g/100 g d.m.)	Sugars (g/100 g d.m.)	TDF (g/100 g d.m.)
MWF	8.0 ± 0.2 c	334.8 ± 1.6 b	14.7 ± 0.2 a	2.36 ± 0.05 a	2.8 ± 0.2 ab	54.0 ± 1.1 b	3.2 ± 0.1 b	18.7 ± 0.9 a
MRF	9.7 ± 0.4 b	335.3 ± 1.3 b	11.0 ± 0.3 b	1.54 ± 0.03 c	1.9 ± 0.1 b	62.0 ± 1.2 ab	8.1 ± 0.3 a	14.6 ± 0.6 ab
MSF	8.7 ± 0.3 c	345.4 ± 1.4 ab	14.6 ± 0.5 a	1.63 ± 0.04 c	2.3 ± 0.3 b	61.0 ± 0.9 ab	3.8 ± 0.1 b	12.4 ± 0.5 b
MBF	9.8 ± 0.4 b	340.2 ± 1.2 ab	13.8 ± 0.4 ab	1.75 ± 0.04 c	3.0 ± 0.4 ab	59.0 ± 0.8 ab	3.3 ± 0.1 b	14.1 ± 0.5 ab
MBWF	10.2 ± 0.5 ab	356.9 ± 1.6 a	13.8 ± 0.4 ab	2.08 ± 0.05 b	2.9 ± 0.1 ab	68.0 ± 1.4 a	1.9 ± 0.0 c	4.4 ± 0.2 d
MSGF	10.5 ± 0.5 ab	353.4 ± 1.4 a	13.4 ± 0.4 ab	1.66 ± 0.04 c	3.4 ± 0.4 a	65.0 ± 0.9 a	3.1 ± 0.1 b	7.5 ± 0.3 c
T530	11.6 ± 0.6 a	331.3 ± 1.1 b	9.6 ± 0.1 c	0.55 ± 0.00 d	1.8 ± 0.1 b	64.5 ± 1.0 a	1.2 ± 0.1 d	10.7 ± 0.4 b

Values are expressed as the mean (n = 2) ± standard deviation. Mean values bearing different letters in the same column denote statistical difference (a > b > c, etc.). MWF—wholemeal wheat flour; MRF—wholemeal rye flour; MSF—wholemeal spelt flour; MBF—wholemeal barley flour; MBWF—wholemeal buckwheat flour; MSGF—wholemeal sorghum flour; T530—plain wheat flour type 530.

**Table 2:** Recalculated farinographic water absorption of flours.

Flour Type	Water Absorption (%)
MWF	87.4 ± 0.7 a
MRF	83.4 ± 0.8 b
MSF	76.4 ± 0.5 c
MBF	70.6 ± 0.4 d
MBWF	63.4 ± 0.3 e
MSGF	63.8 ± 0.3 e
T530	60.6 ± 0.4 f

Values are expressed as the mean (n = 2) ± standard deviation. Mean values bearing different letters in the same column denote statistical difference (a > b > c, etc.). MWF—wholemeal wheat flour; MRF—wholemeal rye flour; MSF—wholemeal spelt flour; MBF—wholemeal barley flour; MBWF—wholemeal buckwheat flour; MSGF—wholemeal sorghum flour; T530—plain wheat flour type 530.

**Table 3:** Water retention capacity (WRC) and sodium carbonate retention capacity (SC-SRC) values of flours.

Flour Type	WRC (%)	SC-SRC (%)
MWF	68.9 ± 0.6 ef	82.9 ± 0.9 e
MRF	128.8 ± 0.9 a	121.6 ± 1.1 a
MSF	73.4 ± 0.6 de	100.9 ± 1.0 c
MBF	80.5 ± 0.8 c	109.4 ± 1.1 b
MBWF	89.0 ± 0.8 b	90.5 ± 0.9 d
MSGF	77.2 ± 0.7 cd	92.5 ± 0.9 d
T530	65.9 ± 0.6 f	87.7 ± 0.9 de

Values are expressed as the mean (n = 2) ± standard deviation. Mean values bearing different letters in the same column denote statistical difference (a > b > c, etc.). MWF—wholemeal wheat flour; MRF—wholemeal rye flour; MSF—wholemeal spelt flour; MBF—wholemeal barley flour; MBWF—wholemeal buckwheat flour; MSGF—wholemeal sorghum flour; T530—plain wheat flour type 530.

**Table 4:** Falling Number and amylographic measurement values of flours.

Flour Type	Falling Number (s)	Initial Gelatinization Temperature (°C)	Final Gelatinization Temperature (°C)	Maximum Viscosity (AU)
MWF	324 ± 1.5 c	63.5 ± 0.4 ab	92.3 ± 0.9 a	805 ± 1.8 d
MRF	200 ± 1.0 e	54.6 ± 0.3 d	72.8 ± 0.7 e	495 ± 1.4 e
MSF	238 ± 1.1 d	58.6 ± 0.3 bcd	80.2 ± 0.8 d	353 ± 1.3 f
MBF	389 ± 1.6 b	61.0 ± 0.5 abc	87.2 ± 0.8 abc	1234 ± 2.4 c
MBWF	918 ± 1.9 a	59.8 ± 0.4 bc	88.7 ± 0.7 ab	3702 ± 3.5 a
MSGF	398 ± 1.6 b	65.6 ± 0.7 a	86.4 ± 0.9 bc	1655 ± 1.6 b
T530	320 ± 1.4 c	56.1 ± 0.5 cd	82.5 ± 0.8 cd	540 ± 1.0 e

Values are expressed as the mean (n = 2) ± standard deviation. Mean values bearing different letters in the same column denote statistical difference (a > b > c, etc.). MWF—wholemeal wheat flour; MRF—wholemeal rye flour; MSF—wholemeal spelt flour; MBF—wholemeal barley flour; MBWF—wholemeal buckwheat flour; MSGF—wholemeal sorghum flour; T530—plain wheat flour type 530.

**Table 5:** Rapid visco-analysis (RVA) starch pasting profiles of flours.

Flour Type	Pasting Temperature (°C)	Peak Time (min)	Peak Viscosity (cP)	Holding Viscosity (cP)	Final Viscosity (cP)	Breakdown (cP)	Setback (cP)
MWF	89.7 ± 1.1 a	5.8 ± 0.3 bc	1282 ± 12 d	761 ± 9 c	2074 ± 22 d	522 ± 5 d	1312 ± 17 cd
MRF	79.9 ± 1.0 bc	5.1 ± 0.2 e	846 ± 9 e	379 ± 4 d	1038 ± 16 f	466 ± 4 e	658 ± 9 e
MSF	86.5 ± 1.2 ab	5.4 ± 0.2 d	887 ± 8 e	378 ± 6 d	1022 ± 11 f	508 ± 6 d	644 ± 8 e
MBF	86.4 ± 1.3 ab	6.1 ± 0.3 a	2670 ± 16 a	1244 ± 11 b	2794 ± 21 c	1426 ± 8 a	1550 ± 19 c
MBWF	74.3 ± 0.9 c	5.9 ± 0.4 b	2729 ± 21 a	2460 ± 23 a	5278 ± 31 a	270 ± 5 f	2818 ± 31 a
MSGF	87.2 ± 1.3 ab	6.1 ± 0.3 a	1914 ± 19 b	1348 ± 14 b	3416 ± 25 b	566 ± 9 c	2068 ± 21 b
T530	76.3 ± 1.1 c	5.6 ± 0.1 c	1487 ± 13 c	644 ± 6 c	1541 ± 15 e	842 ± 1.8 b	896 ± 11 de

Values are expressed as the mean (n = 2). Mean values bearing different letters in the same column denote statistical difference (a > b > c, etc.). MWF—wholemeal wheat flour; MRF—wholemeal rye flour; MSF—wholemeal spelt flour; MBF—wholemeal barley flour; MBWF—wholemeal buckwheat flour; MSGF—wholemeal sorghum flour; T530—plain wheat flour type 530.

## Data Availability

Data is contained within the article.
